# Acute limb ischemia secondary to bullet embolism following a cardiac gunshot wound in a pediatric patient

**DOI:** 10.1016/j.jvscit.2023.101231

**Published:** 2023-05-24

**Authors:** Eliza Ferrari, Tiffany Guard, Vivek Prakash, Michael Shih, Melissa Kirkwood, Michael Siah

**Affiliations:** Division of Vascular Surgery, University of Texas Southwestern Medical School, Dallas, TX

**Keywords:** Acute limb ischemia, BB gun, Bullet embolism, Cardiac trauma, Gunshot wound, Missile embolism, Pediatric

## Abstract

Bullet embolism following a gunshot wound to the heart is a very unusual cause of acute limb ischemia. We report the case of a 3-year-old boy who sustained a penetrating cardiac trauma secondary to an accidental self-inflicted gunshot wound with a BB (ball bearing) gun. The BB pellet entered the left ventricle and embolized into the peripheral circulation, lodging at the bifurcation of the left common femoral artery. This resulted in acute left lower extremity ischemia. The patient was successfully treated by open common femoral artery exploration and foreign body removal.

Acute limb ischemia (ALI) can rarely be caused by missile embolism after a gunshot wound to the chest. We report the case of a pediatric patient who sustained penetrating cardiac trauma secondary to a self-inflicted gunshot wound with a BB (ball bearing) gun. The BB pellet embolized to the bifurcation of the left common femoral artery, resulting in ALI, which was treated by open exploration and foreign body removal. Our patient's family provided written informed consent for the report of his case details and imaging studies.

## Case report

**Fig 1:**
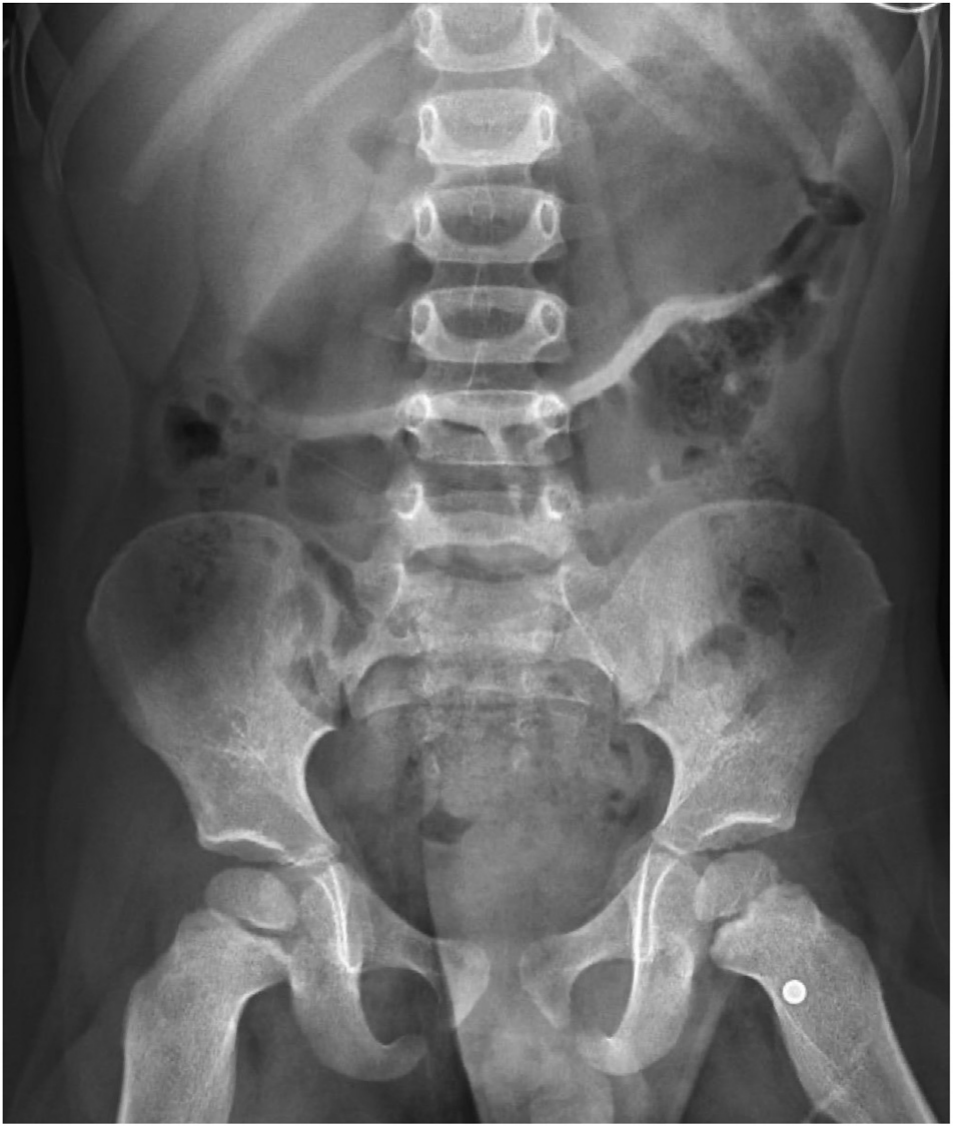
Plain radiograph of the abdomen showing the pellet overlying the left groin.

A 3-year-old boy with no medical history accidentally shot himself in the chest with a BB gun. The patient arrived in the trauma bay 1 hour after the injury. He was hemodynamically stable on arrival with a blood pressure of 97/60 mm Hg, heart rate of 116 bpm, and oxygen saturation of 98% on room air. His Glasgow coma scale score was 15, but he was crying and lethargic. A BB gunshot wound was present at the left parasternal border, and no exit wound was present. The cardiac examination findings were unremarkable. Pulse examination revealed palpable bilateral femoral pulses, palpable right pedal pulses, and nonpalpable left pedal pulses. Both legs were well perfused with intact sensorimotor function. Within 15 minutes of arrival, a chest radiograph was obtained, with unremarkable findings. An abdominal radiograph showed a foreign body overlying the left groin (Fig 1).

**Fig 2:**
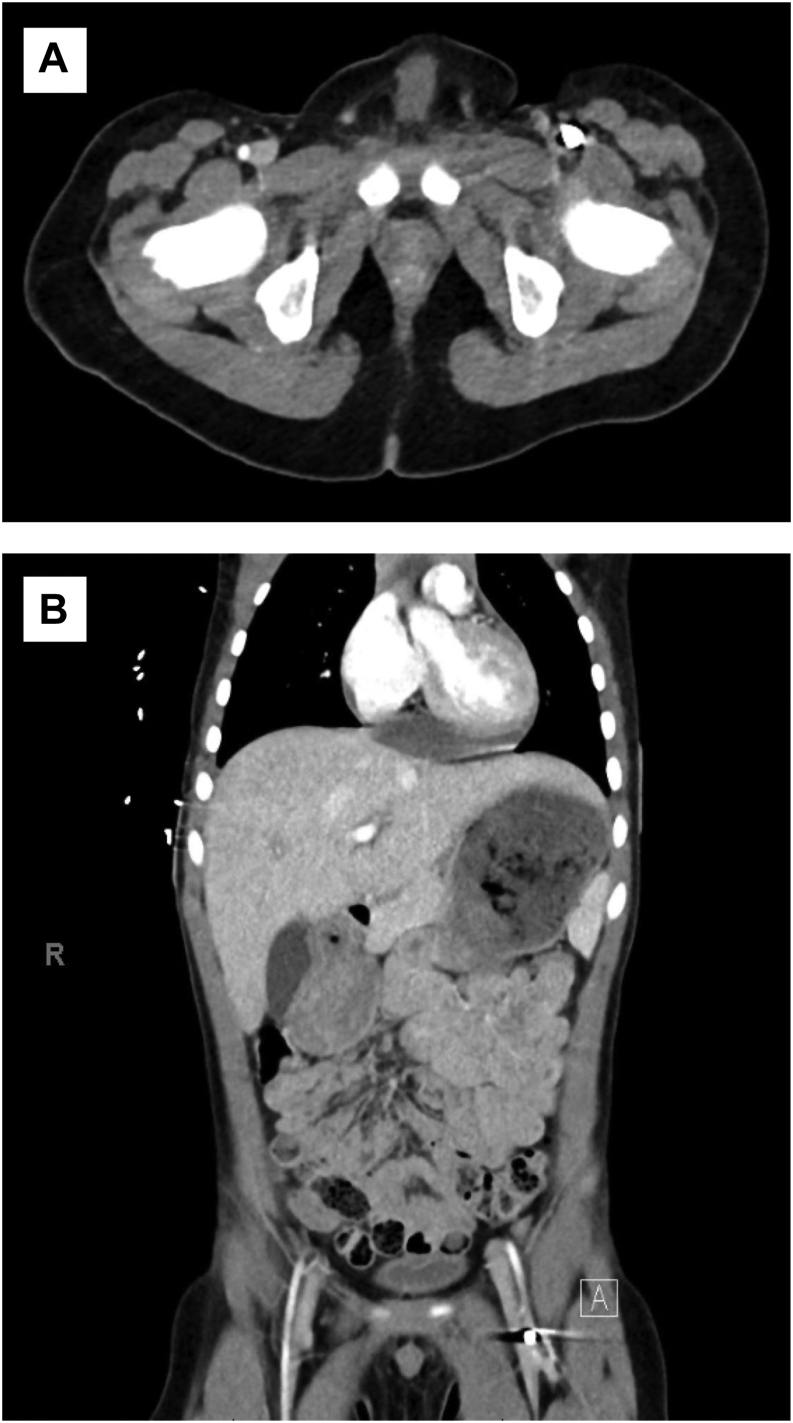
**A,** Axial view of computed tomography (CT) scan of the abdomen and pelvis with intravenous contrast (axial view) showing the pellet in the left common femoral artery (CFA). **B,** Coronal view of CT scan of the abdomen and pelvis with intravenous contrast showing the pellet at the bifurcation of the left CFA to the profunda femoris artery and superficial femoral artery.

Because the patient was stable and the presence of a penetrating cardiac injury was uncertain, computed tomography (CT) of the chest, abdomen, and pelvis with contrast was obtained to evaluate the heart and clarify whether the foreign body was intravascular. The CT scan was completed 1 hour after arrival and demonstrated a penetrating injury to the right ventricle and interventricular septum with a small-volume hemopericardium. A 5-mm intra-arterial foreign body was visualized at the junction of the left profunda femoris and superficial femoral arteries, suggesting that the bullet had passed through the IVS to the left ventricle and subsequently embolized (Fig 2).

The patient developed signs of Rutherford grade IIa ALI of the left leg ∼30 minutes after the CT scan (2.5 hours after the injury). He maintained a palpable left femoral pulse but no arterial Doppler signals were present distally. Venous signals were intact to the foot. Pain and poikilothermia were present but no pallor or sensorimotor loss were noted. We acknowledge that performing a neurovascular examination in young children is challenging. However, we observed our patient moving both lower extremities equally, including the toes, and found that he reacted to a light touch on both feet. The patient was taken to the operating room for repair of the penetrating cardiac injury with concurrent exploration of the left common femoral artery. Approximately 3.5 hours had elapsed between the first clinical manifestations of ALI and restoration of blood flow (6 hours after the injury).

The cardiac surgery team performed a mediastinal exploration, which revealed a bullet wound to the left parasternal chest and a tense pericardium, consistent with cardiac tamponade. A penetrating wound to the right ventricular outflow tract was identified, which was repaired with a single pledgeted stitch. A pleuropericardial window was created, and bilateral chest tubes were placed. At the beginning of the case, the patient was transiently unstable and required vasopressors, but he quickly stabilized after the hemopericardium had been evacuated.

For the vascular portion of the case, a longitudinal incision was made over the left groin for maximum visualization of the common femoral artery (CFA) and to allow for cranial or caudal extension of the incision, if needed. The femoral artery sheath was incised with electrocautery until the CFA, profunda femoris artery (PFA), and superficial femoral artery (SFA) were exposed. Exploration disclosed a metal BB pellet occluding the bifurcation of the CFA; 100 U/kg of heparin was given intravenously. After obtaining proximal and distal control, the arteries were clamped. A transverse arteriotomy was made at the junction of the arteries proximal to the occlusion, and the foreign body was removed. A size 2 Fogarty balloon catheter was inserted into the SFA and PFA without any clot or embolus return. Good back bleeding was present from the PFA and SFA with release of the clamps. The arteriotomy was then closed with multiple interrupted 6-0 Prolene sutures, which were chosen over running sutures to avoid constricting the artery during future growth.

On postoperative day 2, the patient returned to the operating room for repeat mediastinal exploration because of high chest tube output. No active bleeding was identified; however, a clot was removed from the right pleural space. His cardiac surgical course was uncomplicated afterward. From a vascular perspective, the patient recovered as expected and maintained palpable bilateral femoral and pedal pulses throughout the postoperative period. He was discharged on postoperative day 6 after the index operation with a plan for lifelong aspirin therapy (81 mg daily) to reduce the risk of thromboembolic events related to his cardiac and vascular surgeries.

At the clinic follow-up visit 1 month after discharge, the patient was ambulating normally with bilateral palpable femoral and pedal pulses and no pain or sensorimotor loss of the left leg. At that time, a left lower extremity arterial ultrasound scan showed normal vessels with normal waveforms. The repaired left CFA showed a peak systolic velocity of 108 cm/s compared with the right CFA (peak systolic velocity, 90.1 cm/s). The patient also did well from a cardiac perspective. An echocardiogram was performed 1 month postoperatively, which showed trace tricuspid valve insufficiency and normal ventricular systolic function.

## Discussion

Bullet embolism is an unusual cause of ALI and, when secondary to penetrating cardiac trauma, it is an even rarer phenomenon. Our literature review found 16 cases of ALI secondary to bullet embolism after a cardiac gunshot wound (Table).^1^, ^2^, ^3^, ^4^, ^5^, ^6^, ^7^, ^8^, ^9^, ^10^, ^11^, ^12^, ^13^, ^14^, ^15^ We included only those cases with documentation of both a penetrating bullet wound to the heart and signs or symptoms of Rutherford stage IIa, IIb, or III ALI. Our patient is the youngest reported in the literature, with only two other reports involving pediatric patients.^4^^,^^9^ All patients were treated with open embolectomy, except for the patient described by O'Neill,^2^ who had died before treatment of the extremity. In addition, one patient underwent an attempt at endovascular retrieval of the bullet before definitive open treatment.^12^

**Table:** Cases of acute limb ischemia secondary to bullet embolism following cardiac gunshot wound

Investigator	Age, years; sex	Cardiac injury pattern	Embolus	Arterial embolization site
Rubesch,^1^ 1912	28; Male	Unspecified; left side of heart	Bullet	Right common femoral artery
O'Neill,^2^ 1917	Adult; male	Entry to LV	Shell fragment	Left common iliac artery
Saltzstein et al,^3^ 1963	32; Male	Entry to RV, through IVS to LV	0.45 Caliber bullet	Right axillary artery
Neerken et al,^4^ 1964	10; Male	Entry to RV, through IVS to LV	Air gun pellet	Right brachial artery
Katz et al,^5^ 1966	39; Male	Entry to LV	0.32 Caliber bullet	Right common iliac artery
Rodriguez et al,^6^ 1975	19; Female	Entry to RV, through IVS to LV	0.22 Caliber bullet	Right axillary artery
Symbas et al,^7^ 1977	Unknown	Unspecified; right side of heart	Bullet	Left common femoral artery
Symbas et al,^7^ 1977	Unknown	Entry to left AV groove	Bullet	Left superficial femoral artery
Martire et al,^8^ 1978	29; Female	Entry to LV	Bullet	Right common femoral artery
Schowengerdt et al,^9^ 1985	11; Male	Entry to RV, through IVS to LV	5-mm Air gun pellet	Left distal popliteal artery
Harirchi et al,^10^ 2004	36; Male	Entry to RV, through IVS to LV	Bullet	Right axillary artery
Aoun et al,^11^ 2017	25; Male	Entry to LV	7.62 × 39-mm Bullet	Left common femoral artery
Green et al,^12^ 2021	20; Male	Entry to LA	Bullet	Right subclavian artery
Mashayekhi et al,^13^ 2021	21; Male	Entry to LA	9-mm Bullet	Right common iliac artery
AlAttab et al,^14^ 2022	38; Male	Entry to LV	Bullet	Right popliteal artery
Chen,^15^ 2022	26; Male	Entry to LA	Bullet	Left common femoral artery

*IVS,* Interventricular septum; *LA,* left atrium; *LV,* left ventricle; *RA,* right atrium; *RV,* right ventricle.

Approximately 80% of patients who sustain a cardiac gunshot wound die before reaching a hospital.^16^ Of the patients who do survive, diagnosing bullet embolism can be difficult. Hemorrhagic shock precludes a reliable peripheral pulse and sensorimotor examination. Clinical signs of cardiac tamponade (Beck's triad) are not always present, which can delay the diagnosis of a cardiac injury.^8^ A disparity between the number of entrance and exit wounds, radiologic findings that are unexpected given a bullet's trajectory, and evidence of limb ischemia should prompt suspicion for embolization.^17^

Open embolectomy remains the standard of care for bullet embolism. An endovascular approach is less practical, because it introduces the risk of access-site complications, is more time-consuming, and requires specialized and/or expensive equipment. To minimize the ischemia time, open embolectomy should be performed as soon as possible after stabilizing any life-threatening injuries. Distal thrombi are often present and should be evacuated.^7^ In the pediatric population, however, distal thrombosis of the occluded artery is less likely because the normal pediatric vasculature is free from prothrombotic atherosclerotic disease.^18^

Although bullet embolism is rare, certain patterns of injury are more common. Bullet embolism from the peripheral venous circulation to the pulmonary arteries is much more common than bullet embolism from the left heart to the arterial circulation.^10^ Among patients who develop arterial embolism, the bullets are more likely to lodge in the lower extremities owing to the effect of gravity.^16^ A greater probability exists of bullet embolism to the left lower extremity because the left iliac artery forms a more obtuse 30° angle with the bifurcation of the abdominal aorta, and the right iliac artery forms a sharper 45° angle.^8^

Bullet embolism is especially rare in the pediatric population, with only two other reported cases of penetrating cardiac trauma resulting in ALI.^4^^,^^9^ The 10- and 11-year-old boys described in these case reports were also accidentally shot with BB pellets. Considering the size match between the embolus and vessel, small-caliber BB pellets are more likely to lodge in a more proximal location in the pediatric population than in the adult population and are, thus, more likely to cause ALI. Because of their small size, pellet emboli are less susceptible to the effects of gravity and are thus more likely to lodge in the cerebral and upper extremity circulation.^9^

BB and pellet guns are often marketed as toys and are not regulated by gun laws. Parents might not be aware that these weapons can result in serious injury to, or even the death of, a child. Despite their small size, the pellets are discharged with high velocity and can cause penetrating injury to internal organs.^19^ Therefore, the same principles of gun safety recommended for larger firearms should also be applied in the use of BB guns. Further research is needed on the epidemiology of BB gun-related vascular injury in the pediatric population to quantify the effects on public health.

## Conclusions

Bullet embolism from a cardiac gunshot wound leading to ALI is a rare phenomenon. It should be suspected in patients with a penetrating thoracic injury, unusual radiologic findings, and the lack of a corresponding exit wound. We present the case of a 3-year-old boy, who suffered this unusual injury and was treated with open embolectomy of the left CFA. This case demonstrates the dangerous consequences of BB pellet gunshot wounds and highlights the special considerations required for the treatment of bullet embolism in pediatric patients.
